# Supplementary material for: Causal Relationship between Aging and Anorexia Nervosa: A White-Matter-Microstructure-Mediated Mendelian Randomization Analysis
Source: Biomedicines. 2024 Aug 16;12(8):1874. doi: 10.3390/biomedicines12081874 (PMC11351342; doi:10.3390/biomedicines12081874)
Supplement: Supplementary file 1 [file biomedicines-12-01874-s001.zip › Supplementary Figure/Figure S4/Singe SNP plot of MR analysis for TL to ubm-b-1820.pdf]

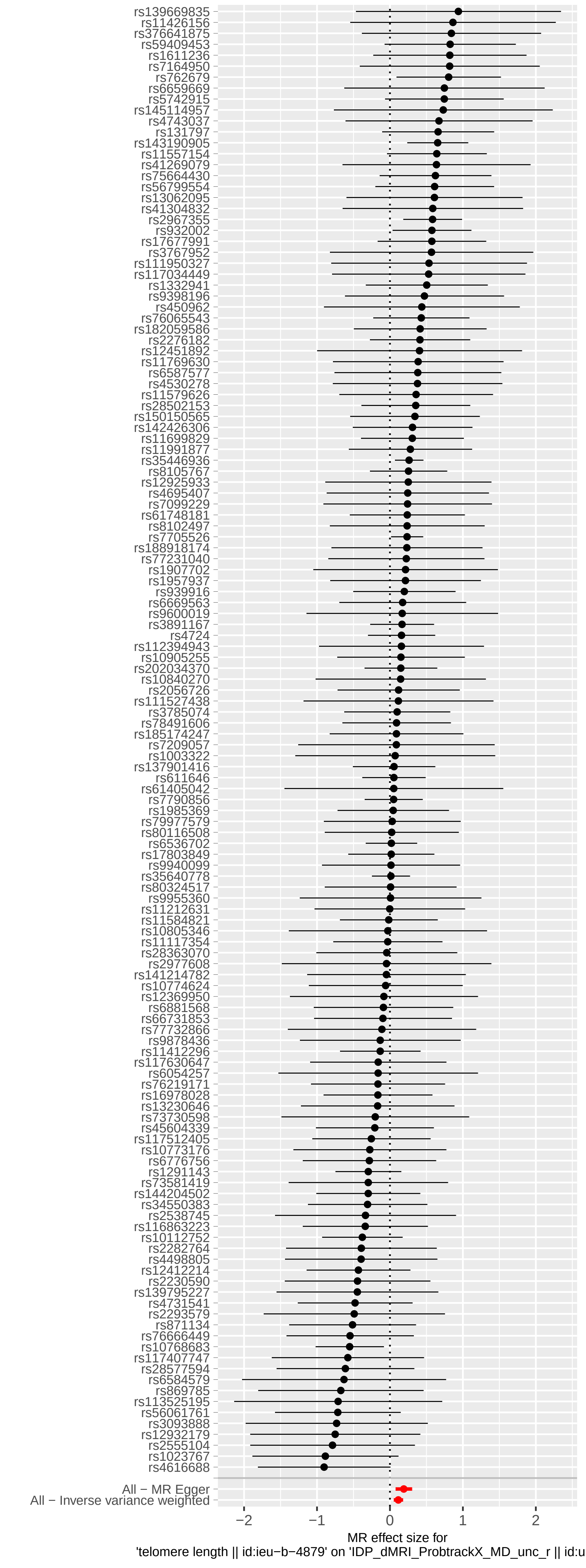
rs139669835
rs11426156
rs376641875
rs59409453
rs1611236
rs7164950
rs762679
rs6659669
rs5742915
rs145114957
rs4743037
rs131797
rs143190905
rs11557154
rs41269079
rs75664430
rs56799554
rs13062095
rs41304832
rs2967355
rs932002
rs17677991
rs3767952
rs111950327
rs117034449
rs1332941
rs9398196
rs450962
rs76065543
rs182059586
rs2276182
rs12451892
rs11769630
rs6587577
rs4530278
rs11579626
rs28502153
rs150150565
rs142426306
rs11699829
rs11991877
rs35446936
rs8105767
rs12925933
rs4695407
rs7099229
rs61748181
rs8102497
rs7705526
rs188918174
rs77231040
rs1907702
rs1957937
rs939916
rs6669563
rs9600019
rs3891167
rs4724
rs112394943
rs10905255
rs202034370
rs10840270
rs2056726
rs111527438
rs3785074
rs78491606
rs185174247
rs7209057
rs1003322
rs137901416
rs611646
rs61405042
rs7790856
rs1985369
rs79977579
rs80116508
rs6536702
rs17803849
rs9940099
rs35640778
rs80324517
rs9955360
rs11212631
rs11584821
rs10805346
rs11117354
rs28363070
rs2977608
rs141214782
rs10774624
rs12369950
rs6881568
rs66731853
rs77732866
rs9878436
rs11412296
rs117630647
rs6054257
rs76219171
rs16978028
rs13230646
rs73730598
rs45604339
rs117512405
rs10773176
rs6776756
rs1291143
rs73581419
rs144204502
rs34550383
rs2538745
rs116863223
rs10112752
rs2282764
rs4498805
rs12412214
rs2230590
rs139795227
rs4731541
rs2293579
rs871134
rs76666449
rs10768683
rs117407747
rs28577594
rs6584579
rs869785
rs113525195
rs56061761
rs3093888
rs12932179
rs2555104
rs1023767
rs4616688
All − MR Egger
All − Inverse variance weighted
−2
−1
0
1
2
MR effect size for
'telomere length || id:ieu−b−4879' on 'IDP_dMRI_ProbtrackX_MD_unc_r || id:u
